# An Evaluation of Cage Amine Sarcophagine Ligands for the Synthesis of Cobalt Radiopharmaceuticals

**DOI:** 10.1021/acs.inorgchem.6c01482

**Published:** 2026-05-27

**Authors:** Hailey A. Houson, Stacey E. Rudd, Solana R. Fernandez, Suzanne E. Lapi, Paul S. Donnelly

**Affiliations:** † Department of Radiology, 9968 University of Alabama at Birmingham, Birmingham, Alabama 35294, United States; ‡ School of Chemistry and Bio21 Molecular Science and Biotechnology Institute, 2281 University of Melbourne, Parkville, Melbourne 3010, Australia

## Abstract

Positron-emitting cobalt-55 (*t*_1/2_ = 17.53 h, β^+^ = 77%, Eγ = 931.1 keV, Iγ = 75%) has the potential to be used for PET imaging and Auger electron-emitting cobalt-58m (*t*_1/2_ = 9.10 h, IC= 100%) has the potential to be used in targeted radionuclide therapy. Sarcophagines are a family of macrobicyclic cage amine ligands that form stable complexes with cobalt­(III). This work investigates the potential of a derivative of sarcophagine with a pendent carboxylic acid functional group, 5-(8-methyl-3,6,10,13,16,19-hexaaza-bicyclo[6.6.6]­icosan-1-ylamino)-5-oxopentanoic acid (known as MeCOSar), to serve as a ligand for cobalt-55. The cobalt­(III) complex, [Co^III^(MeCOSar)]^3+^, was synthesized and characterized as well as a complex where the MeCOSar ligand is conjugated to tumor-targeting peptide Tyr^3^-ocreotate, [Co^III^(SarTATE)]^3+^. The MeCOSar and SarTATE ligands were radiolabeled with cobalt-55 to give [^55^Co]­[Co^III^(MeCOSar)]^*n*+^ and [^55^Co]­[Co^III^(SarTATE)] respectively and their biodistribution was evaluated in non tumor-bearing mice. The tumor uptake of [^55^Co]­[Co^III^(SarTATE)] was then further evaluated in AR42J tumor-bearing mice and compared to the copper-64 complex, [^64^Cu]­[Cu^II^(SarTATE)]. Radiolabeling SarTATE with cobalt-55 requires higher temperatures when compared to copper-64, and [^55^Co]­[Co^III^(SarTATE)] has lower tumor uptake and higher liver uptake in tumor-bearing mice when compared to [^64^Cu]­[Cu^II^(SarTATE)].

## Graphical abstract

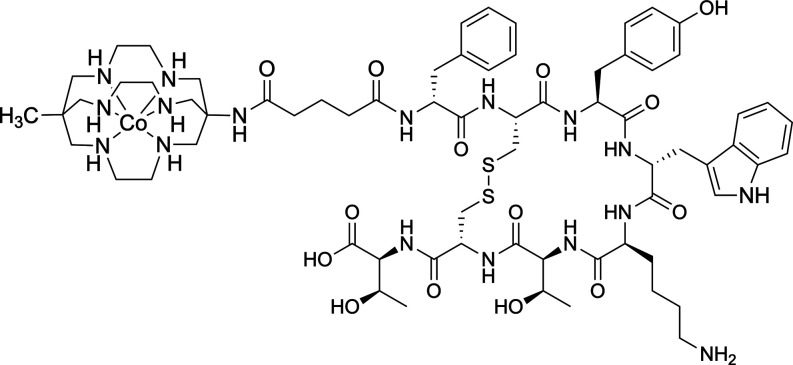

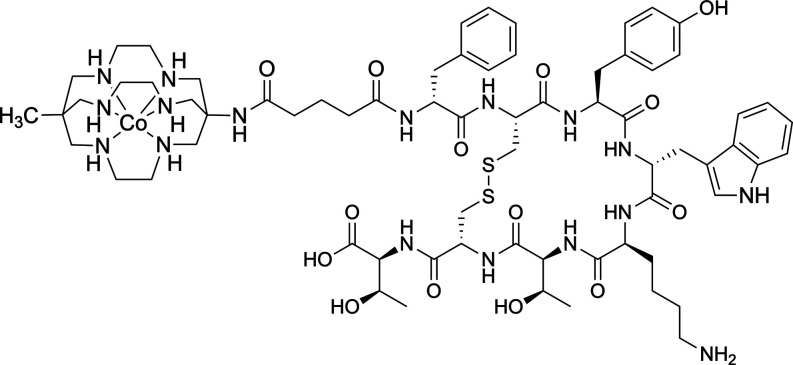

## Introduction

The term “*theranostics*” refers to the concept of using the same or similar molecule for both diagnosis and personalized therapy. In theranostic nuclear medicine, a molecular targeting agent radiolabeled with either of a “matched pair” of radionuclides, is used for diagnostic imaging and targeted therapy.^1–5^ Molecular imaging with positron emission tomography (PET) tracers designed to selectively bind to tumor tissue can identify patients that are suited for follow-on therapy to the same target with α-, β^–^- or Auger-emitting radionuclides.^6,7^ Diagnostic PET imaging can also allow for accurate estimations of the radiation doses delivered to the tumor compared to normal tissues. Metallic radionuclides can be selectively targeted to tumor tissue by their incorporation into coordination compounds that are linked to molecules, such as peptides or antibodies, which selectively bind to receptors that are overexpressed in tumor tissue when compared to normal tissue. Protocols where PET imaging with peptides and small molecules labeled with positron-emitting gallium-68 (*t*_1/2_ = 68 min, Eβ_avg_^+^ = 830 keV, 89%) is used to guide therapy with β-emitting lutetium-177 (*t*_1/2_ = 6.7 days, Eβ_avg_^–^ = 134 keV, 100%) have been translated to the clinic.^8^ It is essential that the metal complex is kinetically inert *in vivo* as dissociation of the metal ion leads to increased uptake of the radionuclide in nontarget tissue. Derivatives of the tetra-aza macrocycle DOTA (1,4,7,10-tetraazacyclododecane-1,4,7,10-tetracetic acid) are often used to coordinate metal radionuclides, such as [^68^Ga]­Ga^III^ and [^177^Lu]­Lu^III^, because the complexes formed are sufficiently inert with respect to dissociation.^2,3^

The somatostatin subtype 2 receptor (SSTR2) is overexpressed in many neuroendocrine tumors and is a validated target for targeted radionuclide therapy.^9^ Modification of DOTA to incorporate an 8-amino acid synthetic analogue of somatostatin, Tyr^3^-octreotate, to give DOTATATE (Figure 1) provides a ligand which retains selectivity for somatostatin receptors that can be radiolabeled with a range of radioactive metal ions. A theranostic approach to treat widely disseminated disease where diagnostic PET imaging with [^68^Ga]­[Ga^III^(DOTATATE)] is used to select patients for targeted radionuclide therapy with [^177^Lu]­[Lu^III^(DOTATATE)] has received clinical approval.^10–14^ This theranostic approach has made a significant contribution to the management of the disease, but the use of two different chemical elements (gallium and lutetium) for imaging and therapy is not ideal as complexes with different metal ions do not always have the same binding and internalization interactions.^15^ Furthermore, there are limitations on modeling dosimetry for the relatively long radioactive half-life of lutetium-177 with the comparatively short-lived gallium-68. The use of different radionuclides of the same element for both imaging and therapy would represent an important advance.

**Figure 1:**
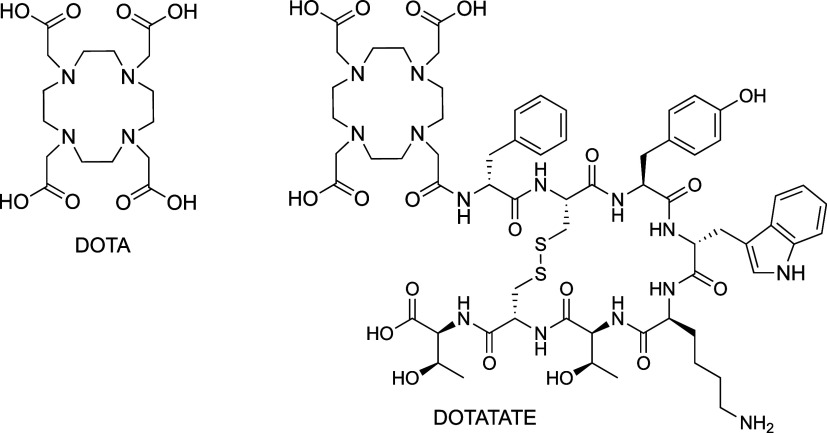
Chemical structures of DOTA and DOTATATE.

One possibility to develop theranostics with paired radionuclides of the same element is to use a single agent labeled with positron-emitting copper-64 (*t*_1/2_ = 12.7 h, β^+^ = 17.4%, E_β_^+^ (mean) = 278 keV) for PET imaging and β-emitting copper-67 for radiotherapy (*t*_1/2_ = 61.9 h, β^–^ = 100%, E_β_^–^(mean) = 141 keV). Recent interest in the development of theranostics that use the copper-64/67 matched pair have focused on the use of a family of macrobicyclic cage amine ligands given the trivial name sarcophagines (Sar = 6,10,13,16,19-hexaazabicyclo[6.6.6]­icosane, Figure 2).^5,16^ These encapsulating cage amine ligands form complexes with copper­(II) radionuclides that are kinetically inert *in vivo*.^17,18^ The modification of the basic Sar framework to incorporate a carboxylate functional group, MeCOSar (Figure 2), allows the attachment of the cage to peptides or antibodies for selective targeting of tumor tissue.^19–22^ A copper-64 complex with a sarcophagine ligand functionalized with Tyr^3^-octreotate, SarTATE (Figure 2), demonstrated high tumor uptake and retention in a xenograft mouse model and in human patients with neuroendocrine neoplasia.^23,24^ The copper-67 complex, [^67^Cu]­[Cu^II^(SarTATE)], has similar therapeutic efficacy to [^177^Lu]­[Lu^III^(DOTATATE)] in a mouse AR42J xenograft model.^25^ A human clinical trial demonstrated the safety and tolerability of [^67^Cu]­[Cu^II^(SarTATE)] in participants with meningioma (NCT03936426) and a trial investigating radionuclide therapy in pediatric patients with high-risk neuroblastoma is in progress (NCT04023331).

**Figure 2:**
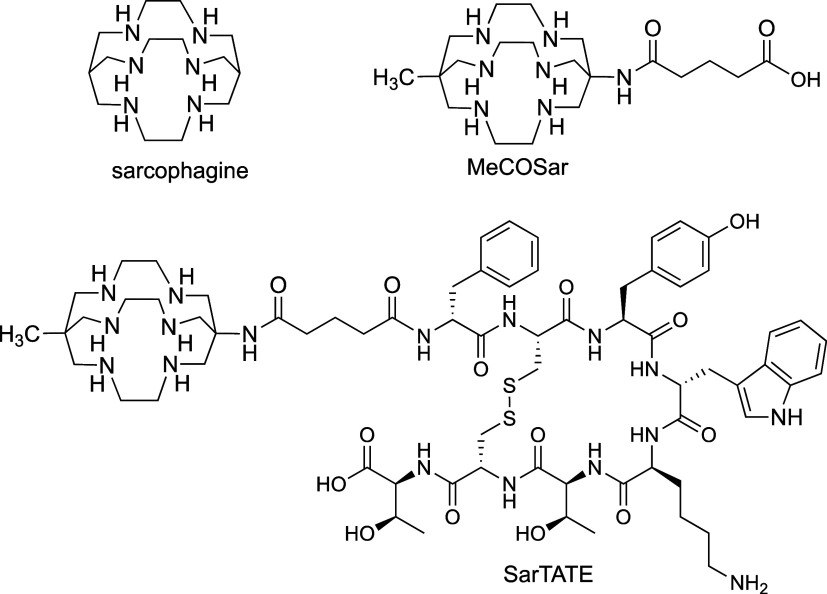
Chemical structures of sarcophagine (sar), MeCOSar and SarTATE.

Ligands derived from sarcophagine also form thermodynamically and kinetically stable complexes with cobalt­(II) and cobalt­(III) and initial synthesis of the ligands involves a cobalt­(III) template.^16,26^ Technological advances have seen significant improvements in the production of a potential theranostic matched pair of cobalt radionuclides, positron-emitting cobalt-55 (*t*_1/2_ = 17.53 h, β^+^ = 77%, Eγ = 931.1 keV, Iγ = 75%) and Auger electron emitting cobalt-58m (*t*_1/2_ = 9.10 h, IC= 100%).^27^ Potential advantages of imaging with cobalt-55 include the high positron yield (77%) and the similar radioactive half-life with the potential therapeutic partner, cobalt-58m.^28^ Potential complications in PET imaging with cobalt-55 are the high-energy γ-emissions 931.1 keV (75%), 1316 keV (7.1%) and 1408.4 keV (16.9%).^29^

As with most radionuclides used for radiopharmaceuticals, the relatively short radioactive half-lives of cobalt-55 and cobalt-58m necessitate ligands that have fast complexation kinetics at moderate temperatures, even when working with low concentrations of metal and ligand (often ≪ 1 μmol L^–1^). It is also essential that the metal complexes are kinetically inert with respect to dissociation and ligand exchange *in vivo*. The two common oxidation states of cobalt, + 2 and +3, typically display dramatically different kinetic lability. The d^7^ cobalt­(II) ion is typically characterized by rapid ligand exchange kinetics whereas the d^6^ cobalt­(III) ion, especially when in the low spin configuration, is kinetically inert due the increased ligand field stabilization energy.^30–32^ Pioneering research of the use of cobalt radionuclides has focused on the use of cobalt­(II) complexes of derivatives of DOTA and NOTA (1,4,7-triazacyclononane-1,4,7-triacetic acid).^28^ The use of different metal ions with different coordination chemistry and the ionic charge of the metal complex has a dramatic effect on binding affinity, biodistribution, clearance pathways and ultimately tumor uptake.^29,33,34^

In this work the sarcophagine derivative, SarTATE, was investigated as a potential ligand for cobalt-55. A significant difference between the use of ligands derived from DOTA or NOTA, such as DOTATATE, and the use of sarcophagine ligands is that the ligand field that results from the N_6_-donor environment of sarcophagine stabilizes the more kinetically inert cobalt­(III) oxidation state relative to cobalt­(II). Encapsulation of cobalt within sarcophagine derived ligands involves initial complexation of the kinetically labile d^7^ cobalt­(II) which is stable in the absence of oxygen or other oxidants, but rapidly oxides to kinetically inert d^6^ cobalt­(III) in ambient concentrations of oxygen.^26^ One of the remarkable features of sarcophagine ligands is that the cobalt­(II) complexes are unusually inert to substitution. There is no exchange with [^60^Co]­[Co^II^(sarcophagine)]^2+^ within 17 h at room temperature and exposure to oxygen results in quantitative oxidation to the cobalt­(III) species.^26^ The cobalt­(III) complexes of sarcophagine-based ligands are also stable *in vivo* and the cobalt remains within the cage amine ligand for at least 24 h following intraperitoneal injection of [^57^Co]­[Co­((NH_2_)_2_sar)]^3+^ in male White Wistar rats.^35^ As expected, this hydrophilic complex is primarily subjected to renal excretion with 67% of the administered radioactivity found in the urine and bladder 2 h after injection and this increased to 90% after 24 h with only 1–2% of radioactivity found in the liver, kidney, muscle, skin, gut and faeces.^35^ Complexation of aqueous buffered mixtures of cobalt­(II) doped with [^57^Co]­Co^II^ (final concentration 10 μM) with a sarcophagine derivative with a pendant aromatic amine demonstrated radiochemical yields of ∼90% within 10 min at room temperature (pH 6–8).^17^ Presumably the initial cobalt­(II) complex oxidizes to cobalt­(III) in the presence of air.

In this study we aimed to investigate the potential of sarcophagine ligands to be used for cobalt-55 radiopharmaceuticals to take advantage of the rapid complexation kinetics characteristic of cobalt­(II) and sarcophagine ligands followed by oxidation to substitutionally inert cobalt­(III) complexes. The complexation of SarTATE with cobalt-55 and copper-64 was investigated and the tumor uptake, biodistribution and clearance of [^55^Co]­[Co­(SarTATE)] with the metal ion presumably in +3 oxidation state compared directly to [^64^Cu]­[Cu­(SarTATE)] where the metal ion is in the +2 oxidation state. During the course of this work the radiolabeling of (NH_2_)_2_sar and a derivative of (NH_2_)_2_sar functionalized with a molecule designed to bind to the neurotensin receptor with [^55^Co]­Co^III^ was published.^36^

## Methods

### General Experimental

All reagents and solvents were obtained from standard commercial sources and unless otherwise stated were used as received. NMR spectra were recorded with a Varian FT-NMR 500 spectrometer (Varian, California USA). ^1^H NMR spectra were acquired at 500 MHz and ^13^C NMR spectra were acquired at 125.7 MHz. All NMR spectra were recorded at 25 °C and all ^13^C NMR were obtained as proton decoupled ^13^C NMR, unless otherwise stated. The reported chemical shifts (in parts per million) are referenced relative to residual solvent signal, except for D_2_O solutions which were spiked with acetone and referenced accordingly (^1^H δ 2.08 ppm, ^13^C δ 30.89 ppm). ESI-MS were recorded on a Thermo Scientific Exactive Plus OrbiTrap LC/MS (Thermo Fisher Scientific, Massachusetts, USA) and calibrated to internal references. Analytical reverse phase HPLC of nonradioactive samples was performed on an Agilent 1200 Series using a Phenomenex Kinetex 5 μm C18 100 Å 150 mm × 4.6 mm column, with a gradient of 0–100% solvent B over 25 min (solvent A: 0.1% TFA in H_2_O, solvent B: 0.1% TFA in CH_3_CN). Absorbance spectra were obtained on a Model UV-1650 PC spectrophotometer (Shimadzu, Kyoto, Japan). Cyclic voltammetry was performed using a PGSTAT100 electrochemical workstation (Metrohm Autolab, Utrecht, The Netherlands) and GPES V4.9 software. Measurements were conducted using a glassy carbon working electrode, a platinum wire as the auxiliary electrode, and a Ag/AgCl reference electrode. All measurements were carried out in aqueous phosphate buffered saline (pH 7.4). The working electrode was polished using 0.3 mm alumina with water on a felt pad. Measurements were performed under an argon atmosphere and redox potentials were reported relative to a standard hydrogen electrode with potassium ferricyanide K_3_[Fe­(CN)_6_] internal standard (*E* ^0^′ = 0.40 V vs SHE).

### Synthesis of [^nat^Co]­[Co^III^(MeCOSar)]­Cl_3_.3H_2_O

[Co^III^((CH_3_)­(NH_3_)­sar)]­Cl_4_ (1.96 g, 3.80 mmol) was stirred in methanol (10 mL, anhydrous), cooled to 0 °C and triflic acid (1.5 mL, 17 mmol) was added dropwise under an atmosphere of nitrogen. The mixture was allowed to warm to room temperature and stirred for a further 1 h before excess hydroch acid was removed by N_2_ flow. The resulting suspension was filtered, washed with methanol and diethyl ether, and dried under vacuum to give a light orange powder. The triflate salt (597 mg, 0.616 mmol) was then taken up into dimethylacetamide (10 mL, anhydrous) and DIPEA (0.2 mL, 1.143 mmol) was added, followed by glutaric anhydride (143 mg, 1.25 mmol). The mixture was heated at 80 °C for 2 h, cooled to room temperature and then diluted with water (100 mL). The solution was applied to an SP Sephadex C-25 cation exchange column (Na^+^ form). The column was eluted with sodium citrate solution (0–0.05 M). The fraction containing the desired complex was loaded directly onto a DOWEX 50W × 2 cation exchange column (H^+^ form), washed with water and HCl (1 mol L^–1^), then eluted with HCl (3–4 mol L^–1^). The solvent was removed under reduced pressure and the residue recrystallized by slow evaporation of H_2_O to give [Co^III^(MeCOSar)]­Cl_3_·3H_2_O as orange crystals (262 mg, 11%). ESIMS: [M-H]^2+^ *m*/*z* (calculated) = 242.6257, *m*/*z* (observed) = 242.6257; [M+2MeCN]^3+^ *m*/*z* (calculated) = 189.4372, *m*/*z* (observed) = 189.4374; [M+MeCN]^3+^ *m*/*z* (calculated) = 175.7617, *m*/*z* (observed) = 175.7618. ^1^H NMR (500 MHz, D_2_O) δ (ppm) 3.39 (d, *J* = 13.7 Hz, 3H, CONHCC*H*_2_), 3.24 (dd, *J* = 23.8, 11.5 Hz, 6H, NHC*H*_2_C*H*_2_NH), 2.94 (d, *J* = 13.9 Hz, 3H, CH_3_CC*H*_2_), 2.78 (d, J = 13.8 Hz, 3H, CONHCC*H*_2_), 2.70–2.51 (m, 6H, NHC*H*_2_C*H*_2_NH), 2.33 (d, *J* = 13.9 Hz, 3H, CH_3_CC*H*_2_), 2.26 (t, *J* = 7.3 Hz, 2H, NHCOC*H*_2_), 2.14 (t, *J* = 7.3 Hz, 2H, COOHC*H*_2_), 1.71 (p, *J* = 7.3 Hz, 2H, COOHC*H*_2_CH_2_), 0.80 (s, 3H, C*H*_3_). ^13^C NMR (126 MHz, D_2_O) δ (ppm) 177.8 (*C*OOH), 176.0 (*C*ONH), 58.2 (CONH*C*CH_2_), 54.6 and 54.5 (CH_3_C*C*H_2_ and CONHC*C*H_2_), 51.1 and 51.0 (NH*C*H_2_ *C*H_2_NH), 42.1 (CH_3_ *C*CH_2_), 34.9 (COOH*C*H_2_), 32.8 (NHCO*C*H_2_), 20.2 (COOHCH_2_ *C*H_2_), 19.2 (*C*H_3_). Elemental analysis for C_20_H_47_N_7_O_6_Cl_3_Co, calculated: C 37.13, H 7.32, N 15.16; found: C 36.96, H 7.34, N 14.97.

Crystals suitable for single crystal X-ray crystallography were grown from a solution of the complex dissolved in D_2_O. Diffraction data were collected at low temperature (*T* = 100 K) with Mo Kα radiation (λ = 0.71073 Å). The structure was solved using SHELXT and refinement was carried out with SHELXL employing full-matrix least-squares on *F* ^2^ using the Olex2 software package.^37–39^ Crystal data: for C_20_H_51_Cl_3_CoN_7_O_8_ (*M* = 682.95 g/mol): triclinic, space group

P1̅, *a* = 9.02300(1) Å, *b* = 12.93730(1) Å, *c* = 13.92910(1) Å, α = 75.4230(1)°, β = 78.9930(1)°, γ = 84.0740(1)°, *V* = 1542.09(3) Å^3^, *Z* = 2, *T* = 100.00(10) K, μ­(Mo Kα) = 0.869 mm^–1^, *Dcalc* = 1.471 g/cm^3^, 87,405 reflections measured (4.606° ≤ 2Θ ≤ 102.588°), 33,000 unique (*R*_int_ = 0.0351, *R*_sigma_ = 0.0410) which were used in all calculations. The final *R*_1_ was 0.0346 (I > 2σ­(I)) and *w*R**_2_ was 0.1190 (all data). CCDC 2539449.

SarTATE was synthesized using standard solid phase peptide synthesis as described previously,^23^ with the exception of the final cyclization step which used 2,2′-dithiobis­(5-nitrodipyridine) instead of the previously reported 2,2′-dithiodipyridine.

[^nat^Co]­[Co^III^(SarTATE)] was prepared using the same procedure as SarTATE, using [Co^III^(MeCOSar)]­Cl_3_.3H_2_O in lieu of Boc_4–5_MeCOSar in the final coupling step to give a pale orange powder (4 mg). ESIMS: [M]^3+^ *m*/*z* (calculated) = 505.5542, *m*/*z* (observed = 505.5560), [M-H]^2+^ *m*/*z* (calculated) = 757.8277, *m*/*z* (observed) = 757.8278

### Radiolabeling with Cobalt-55 and Copper-64

Copper-64 and cobalt-55 were produced by the UAB Cyclotron Facility as previously described.^34^

SarTATE was dissolved in PBS at pH 8 at 5 mg/mL. [^55^Co]­[Co^II^] (22.2 MBq, 600 μCi) was neutralized with PBS and NaOH and added to SarTATE (12.5 μg, 16.5 nmol). The reaction mixture was heated at 90 °C for 30 min. The reaction mixture was analyzed by radio-TLC using iTLC paper and diethylenetriaminepentaacetic acid (DTPA) (50 mM in water). Under these conditions [^55^Co]­[Co^II^(DTPA)]^3–^ moves with the solvent front, and [^55^Co]­[Co^III^(SarTATE)] remains at the baseline.

Copper-64 (46.25 MBq, 1.25 mCi) in 0.1 M HCl was neutralized with PBS and NaOH and combined with SarTATE (12.5 μg, 16.5 nmol) in PBS (50 μL, pH 8). The reaction mixture was heated for 30 min at 50 °C. Analysis of the reaction mixture by radio-TLC was performed using iTLC plates and developed in DTPA in water (50 mM). Under these conditions, free [^64^Cu]­[Cu^II^(DTPA)]^3–^ moves with the solvent front and [^64^Cu]­[Cu^II^(SarTATE)] remains at the baseline.

To estimate the LogD, radiolabeled SarTATE was added to a test tube with octanol (2 mL) and of water (2 mL). The mixture was vortexed at maximum speed for 2 min and then the phases were allowed to separate (5 min). Samples were taken from each phase and activity was counted on a γ counter (PerkinElmer, Greenville, NC, USA).

To assess the stability of radiolabeled SarTATE, samples of the radiolabeled compounds were combined with PBS and at designated times, 100 μL was removed for HPLC. HPLC was performed on an Agilent 1260 system (Santa Clara, CA, USA) with an in-line Flow-RAM NaI detector (LabLogic, Brandon, FL, USA) using a Phenomenex column (Kinetex EVO 150 mm × 4.6 mm) and a gradient of 5–50% ACN over 25 min.

Serum stability was assessed by combining radiolabeled SarTATE with human serum and incubated at 37 °C. At indicated time points, aliquots of the mixture were combined with methanol to precipitate the serum proteins. The supernatant was then analyzed by reverse-phase HPLC.

MeCOSar was dissolved in PBS at a concentration of 5 mg/mL and combined with cobalt-55 or copper-64 at a concentration of 13 MBq/umol. The solution was heated at 50 °C for 30 min and the reaction product was developed using Si-60 plates using 50 mM DTPA solution. The respective DPTA complexes with cobalt and copper move with the solvent front and labeled product remains at the baseline.

### PET Imaging and Biodistribution Studies in Mice

#### SarTATE Study

All animal studies were approved by the Institutional Animal Care and Use Committee at the University of Alabama at Birmingham and were compliant with national animal welfare policies and guidelines. Female athymic nude mice were purchased from Charles River (Charles River, Wilmington, MA, USA). Tumor implantation consisted of an injection of 600,000 AR42J cells in PBS in the right shoulder 3–4 weeks before the study. On the first imaging day, 1 μg [^55^Co]­[Co^III^(SarTATE)] (0.96 MBq, 26 μCi) or 1 μg [^64^Cu]­[Cu^II^(SarTATE)] (2.25 MBq, 61 μCi) were injected via the retroorbital sinus. PET/CT data was acquired on a GNEXT PET/CT (Sofie Biosciences, Dulles, VA, USA) at 1h (15 min PET), 4 h (20 min PET), and 24 h (25 min PET) after injection followed by CT for anatomical reference. After the 24 h imaging time point, the mice were euthanized and organs were collected, weighed and counted on a γ counter (Hidex, Turku, Finland) for biodistribution analysis. Total organ mass for blood, fat, muscle, and bone was assumed as 7.78, 15, 41, and 10.9% of the mouse’s body mass.

#### MeCOSar Study

[^55^Co]­[Co^III^(MeCOSAr)] or [^64^Cu]­[Cu^II^(MeCOSAr)] was injected into BALB/c mice via the tail vein. Mice were injected with either ∼4.5 MBq (80 μg, 187 nmol) of [^55^Co]­[Co^III^(MeCOSAr)] or ∼4.0 MBq (80 μg, 187 nmol) of [^64^Cu]­[Cu^II^(MeCOSAr)]. Mice were imaged immediately post injection (30 min PET), at 4 h (20 min PET), and at 24 h (25 min PET) followed by a CT for anatomical reference. After the 24 h imaging time point, mice were euthanized and organs were harvested for biodistribution analysis. Free cobalt imaging was performed by injecting [^55^Co]­Co^II^Cl_2_ (3.7 MBq in saline) in BALB/c mice via the tail vein. Imaging was performed at 24 h post injection and biodistribution was assessed after imaging.

### Statistical Analysis

All comparisons presented were done using an ordinary 2-way ANOVA and a Šídák’s multiple comparisons test.

## Results

The cobalt complex of nonradioactive [^nat^Co]­[Co^III^(MeCOSar)]^3+^ (from here referred to as [Co^III^(MeCOSar)]^3+^) was prepared to investigate the coordination chemistry and electrochemistry of the complex as well as provide a nonradioactive reference standard to help characterize the cobalt-55 complex. Acylation of [Co­((Me)­(NH_2_)­sar)]^3+^ with glutaric anhydride in dimethylacetamide in the presence of diisopropylethylamine followed by purification by ion-exchange chromatography allowed isolation of [Co^III^(MeCOSar)]^3+^ as its chloride salt (Figure 3). This approach was adapted from the synthesis of [Cu^II^(MeCOSar)]^2+^ and other acylated derivatives.^21,23,40,41^ The coordination of the secondary amine nitrogen atoms to cobalt­(III) effectively reduces their reactivity to acylation and promotes selective acylation of the primary amine of [Co­((Me)­(NH_2_)­sar)]^3+^.^19,41^ Analysis of the complex by electrospray mass spectrometry revealed the main molecular ion at *m*/*z* = 242.6257 ([M-H]^2+^ ion). Analysis of the diamagnetic d^6^ Co­(III) complex by ^13^C NMR spectroscopy revealed the expected 12 resonances consistent with the pseudo *C*_3_ symmetry. The resonance for the methyl carbon is at δ 19 ppm and the resonances for the carbon atoms of the amide and carboxylate functional groups at δ 178 ppm and δ 176 ppm, respectively. The ^1^H NMR spectrum consists of three distinct resonances from the glutaric acid acylation, along with eight well resolved resonances for the methylene protons, each integrating to three (or in some cases reported as 6 for overlapping signals), which is representative of the magnetic inequivalence of the CH_2_ protons once the ligand is bound to cobalt.

**Figure 3:**
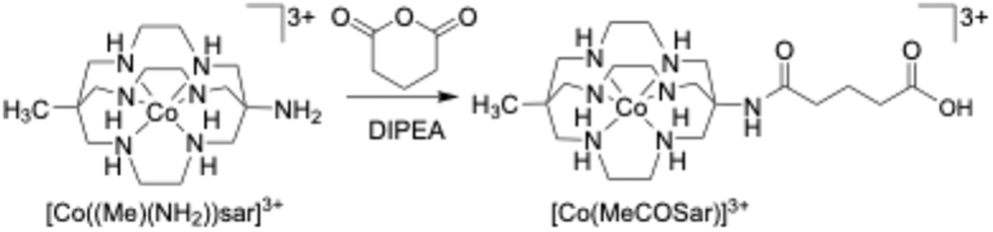
Synthesis of [Co^III^(MeCOSar)]^3+^.

Crystals of [Co^III^(MeCOSar)]^3+^ suitable for analysis by X-ray crystallography (Figure 4a) were isolated from a D_2_O solution of the complex that was used for analysis by NMR. The cobalt­(III) ion is in the expected distorted octahedral environment. The Co–N bonds lengths range from 1.9728(4) Å (Co–N5) to 1.9816(4) Å (Co–N6) and are similar to the Co–N bond lengths in [Co­(NH_3_)_2_sar]­Cl_5_.4H_2_O^42^ and carboxymethylated derivatives, [Co­((NH_2_CH_2_CO_2_H)_2_sar)]^5+^.^19^ Both the Δ- and Λ- enantiomers are present in the unit cell and the C–C bonds of the five membered chelate rings are approximately parallel to the *C*_3_ axis of the macrobicylic sarcophagine consistent with a *lel*_3_ conformation.^16,19,41,43^ The lattice involves an extensive hydrogen-bonding network and the *lel*_3_ conformation is associated with chelation of the three chloride ions by hydrogen bonding interactions with two secondary amines from three separate “limbs” of the cage ligand (Figure 4b). This chelation of hydrogen-bond acceptors is commonly encountered in cage amine complexes with the *lel*_3_ conformation.^19^ The oxygen of the amide functional group is involved with a hydrogen-bond interaction with one of the waters of crystallization (O1­(amide)···O7­(H_2_O)), and this water has a further hydrogen-bond to a second molecule of water (O7­(H_2_O))···(O8 (H_2_O)) which also has a close contact to a chloride counterion (08­(H_2_O)···Cl3) (Figure 4b). The other three solvent water molecules are also involved in close-contacts with chloride anions.

**Figure 4:**
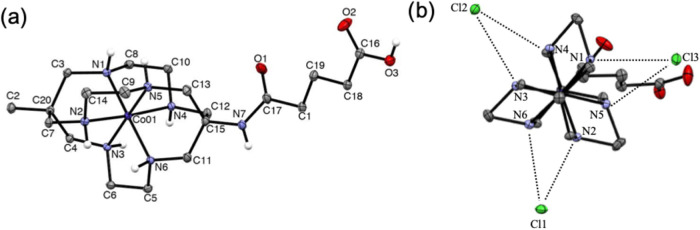
(a) An ORTEP-3 representation (50% ellipsoids) of the cation present in the X-ray crystal structure [Co^III^(MeCOSar)]­Cl_3_.5H_2_O. Anions, solvent and hydrogen atoms bound to carbon omitted for clarity. (b) View down pseudo 3-fold axis to highlight chelation of chloride by NH donors and hydrogen bonding (hydrogen atoms and solvent omitted for clarity).

The orange [Co^III^(MeCOSar)]^3+^ complex retains the *lel*_3_ conformation in solution as absorption spectroscopy in water reveals a λ_max_ = 471 nm consistent with the ^1^A_1g_ → ^1^T_1g_ transition (Figure 5a). Addition of an excess of zinc dust to the solution reduced the Co­(III) center to Co­(II) leading to a color change from orange to pale green/gray. The orange color returned upon exposure to air. The electrochemistry of the complex [Co^III^(MeCOSar)]^3+^ was also investigated by cyclic voltammetry (Figure 5b). In aqueous phosphate buffered saline (pH 7.4) the complex undergoes a one electron quasi-reversible redox process at *E*_1/2_ = −0.428 V vs SHE (Δ*E* = 75 mV), which is attributed to a Co­(III/II) process. This potential is quoted versus SHE by an internal reference of K_3_[Fe­(CN)_6_], (*E*_1/2_ = 0.40 V vs SHE; Δ*E* = 82 mV).

**Figure 5:**
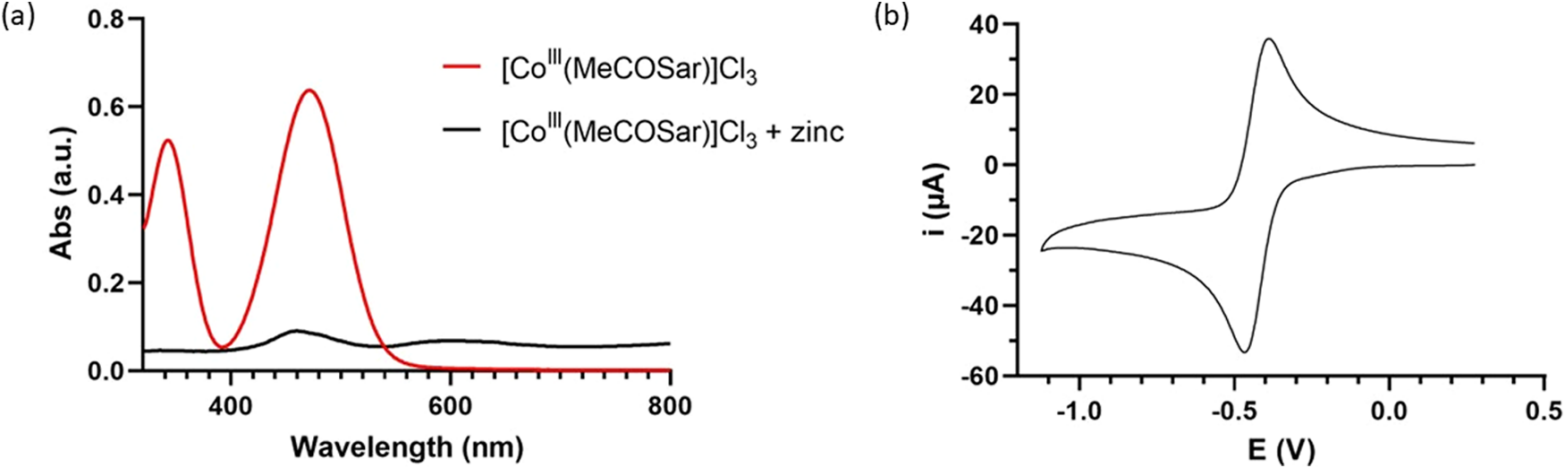
(a) UV Vis spectroscopy of (red) [Co^III^(MeCOSar)]­Cl_3_.3H_2_O, 4.5 mM in H_2_O (λ_max_ = 342 nm, 471 nm), and (black) following addition of zinc dust. (b) Cyclic voltammogram of [Co^III^(MeCOSar)]­Cl_3_ (4.6 × 10^–3^ mol L^–1^) measured in phosphate buffered saline (pH 7.4), at a glassy carbon electrode, scan rate = 50 mV s^–1^, potentials quoted versus SHE to an internal reference of K_3_[Fe­(CN)_6_].

Nonradioactive [Co^III^(SarTATE)] was prepared using solid phase peptide synthesis to assist in characterization of the radioactive analogue, [^55^Co]­[Co^III^(SarTATE)]. Conversion of the chloride salt of [Co^III^(MeCOSar)]^3+^ to a triflate salt provides a form of the complex that is soluble in dimethylformamide and suitable for the synthesis of [Co­(SarTATE)]^3+^ on resin, where the final coupling step involves addition of the metal complex. The cobalt complex, [Co­(SarTATE)]^3+^, was characterized by HPLC and ESI-MS with the complex being detected in the electrospray-MS as the [M]^3+^ *m*/*z* 505.5560 (Figure 6).

**Figure 6:**
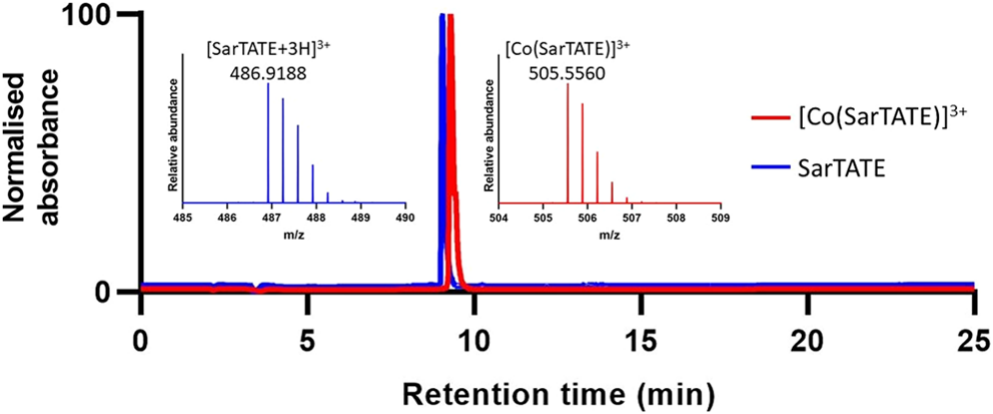
HPLC trace of (red) [Co­(SarTATE)]^3+^ complex and (blue) SarTATE, with (insets) ESI-MS spectra showing the respective 3+ ions.

Formation of [^55^Co]­[Co^III^(SarTATE)] was monitored by analysis of the reaction mixtures by radio-thin layer chromatography (radio-TLC) (Figure 7A). When using an aqueous mobile phase containing diethylenetriaminepentaacetic acid (DTPA) (50 mM), [^55^Co]­[Co^II^(DTPA)]^3–^ moves with the solvent front while [^55^Co]­[Co^III^(SarTATE)] is retained at the baseline (Figure 7). Initial investigations of the use of buffers with different pH values (pH 6–9) indicated that the best radiochemical yields were obtained in phosphate buffered saline (PBS) at pH 8 (Figure 7B). Experiments were performed to determine potential maximal loading of cobalt-55 with 50, 25, and 10 μCi (1.8, 0.9, and 0.37 MBq) of activity per μg of SarTATE and heated to 50 °C resulted in modest radiochemical yields (Figure 7C). When heated to 80 °C the radiochemical yield increased to >95% at 10 μCi/μg (0.37 MBq/μg) (Figure 7C). [^64^Cu]­[Cu^II^(SarTATE)] was also prepared for direct comparisons with [^55^Co]­[Co^III^(SarTATE)]. Analysis of mixtures of either [^55^Co]­[Co^III^(SarTATE)] or [^64^Cu]­[Cu^II^(SarTATE)] in phosphate buffered saline (pH 7.4) by radio-HPLC confirmed that both complexes are stable for up to 24 h. Consistent with earlier reports [^64^Cu]­[Cu^II^(SarTATE)] is stable in the presence of human serum for at least 24 h, but [^55^Co]­[Co^III^(SarTATE)] incubated in human serum showed some decomposition, with ∼75% of the complex still intact after 24 h (Figure 7D).

**Figure 7:**
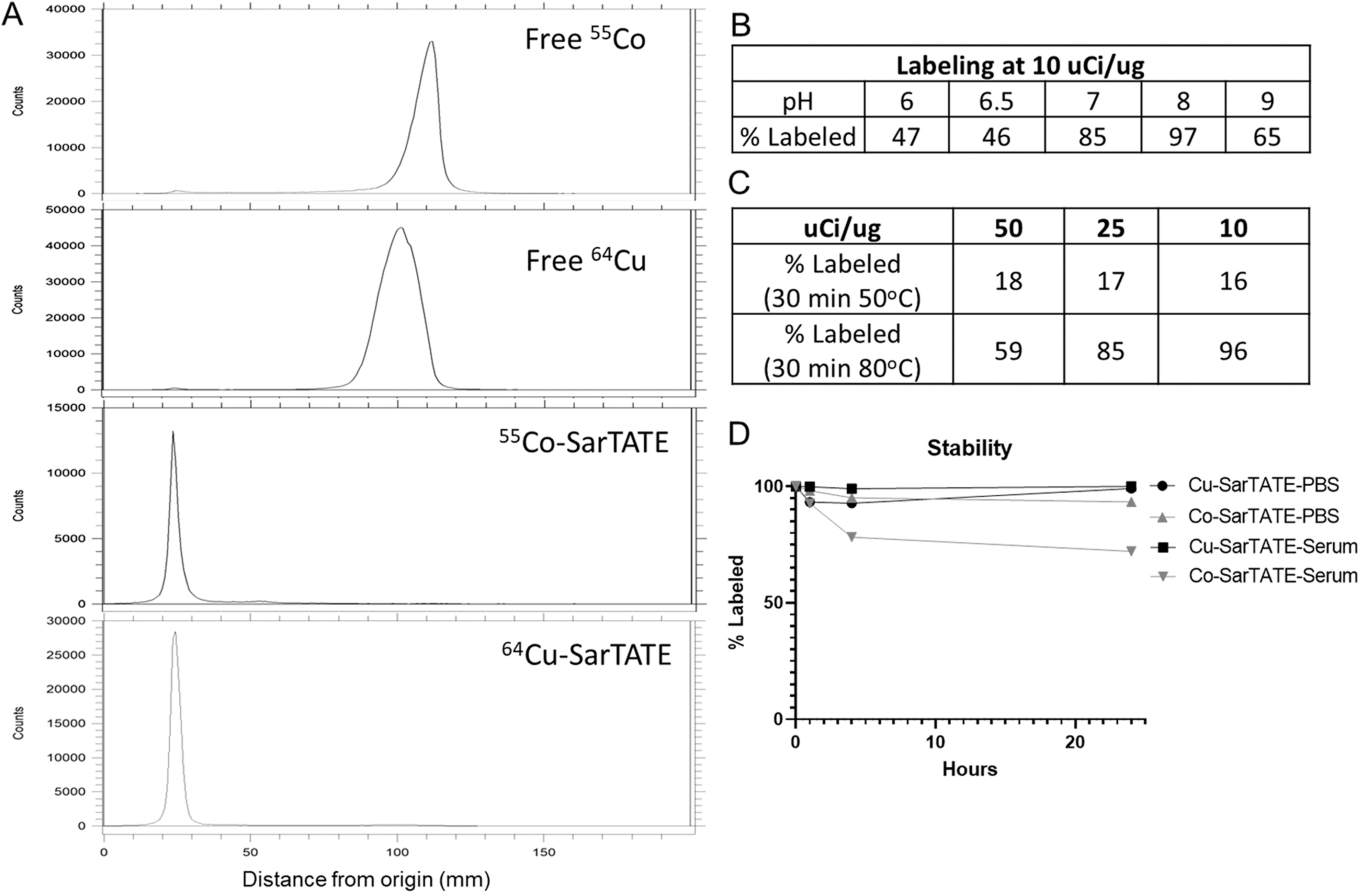
Radiolabeling and stability of [^55^Co]­[Co^III^(SarTATE)]. (A) Radio-TLC chromatograms of free metal and SarTATE radiolabeled with ^55^Co and ^64^Cu. (B) Effect of pH modulation on radiochemical yield of [^55^Co]­[Co^III^(SarTATE)]. (C) Effect of incubation temperature on radiochemical yield of [^55^Co]­[Co^III^(SarTATE)]. (D) Stability of [^55^Co]­[Co^III^(SarTATE)] and [^64^Cu]­[Cu^II^(SarTATE)] in PBS or serum assessed by Radio-HPLC.

The distribution coefficient between octanol and water (Log *D*) is often used to estimate the relative lipophilicity of compounds. The log *D* values of [^55^Co]­[Co^III^(SarTATE)] (log *D* −1.44) and [^64^Cu]­[Cu^II^(SarTATE)] (log *D* −1.42) are similar to each other and consistent with a relatively hydrophilic compound.

PET images were acquired at 1 h, 4 and 24 h after administration of either [^55^Co]­[Co^III^(SarTATE)] (0.96 MBq, 1 μg, 0.69 nmol) or [^64^Cu]­[Cu^II^(SarTATE)] (2.25 MBq, 1 μg, 0.69 nmol) injected via the retroorbital sinus followed by CT imaging for anatomical reference. Inspection of the whole body PET images revealed that [^55^Co]­[Co^III^(SarTATE)] showed high levels of uptake in the liver which was apparent at all time points imaged (Figure 8A). Tumor accumulation was faint, but observable at all time points. [^64^Cu]­[Cu^II^(SarTATE)] showed less liver accumulation and clear tumor accumulation at all time points (Figure 8B). SUV analysis of PET images show that at the 24 h PI time point, the mean tumor SUV was 0.21 ± 0.05 for [^55^Co]­[Co^III^(SarTATE)] and 0.64 ± 0.08 for [^64^Cu]­[Cu^II^(SarTATE)] (Figure 8C,8D). Mean SUV for the liver was 2.7 ± 0.4 for [^55^Co]­[Co­(SarTATE)] and 0.9 ± 0.2 for [^64^Cu]­[Cu­(SarTATE)]. The animals were euthanized 24 h after injection and the radioactivity in the tumor and organs was quantified and represented as the % of injected activity per gram of tissue (%IA/g) (Figure 8E). For [^55^Co]­[Co^III^(SarTATE)] the tumor uptake was 1.4 ± 0.3%IA/g with a relatively high degree of liver uptake 16.4 ± 1.8%IA/g. Using [^64^Cu]­[Cu^II^(SarTATE)] tumor uptake was significantly higher at 4.2%IA/g (*P* < 0.001) and liver uptake significantly lower at 4.3 ± 1.2%IA/g (*P* < 0.0001) (Figure 8E). While the difference was not significant, the amount of [^64^Cu]­[Cu^II^(SarTATE)] remaining in blood at 24 h was 1.8 ± 0.5%IA/Organ compared with [^55^Co]­[Co^III^(SarTATE)] at 0.3 ± 0.1%IA/Organ (Figure 8F) (*P* = 0.06).

**Figure 8:**
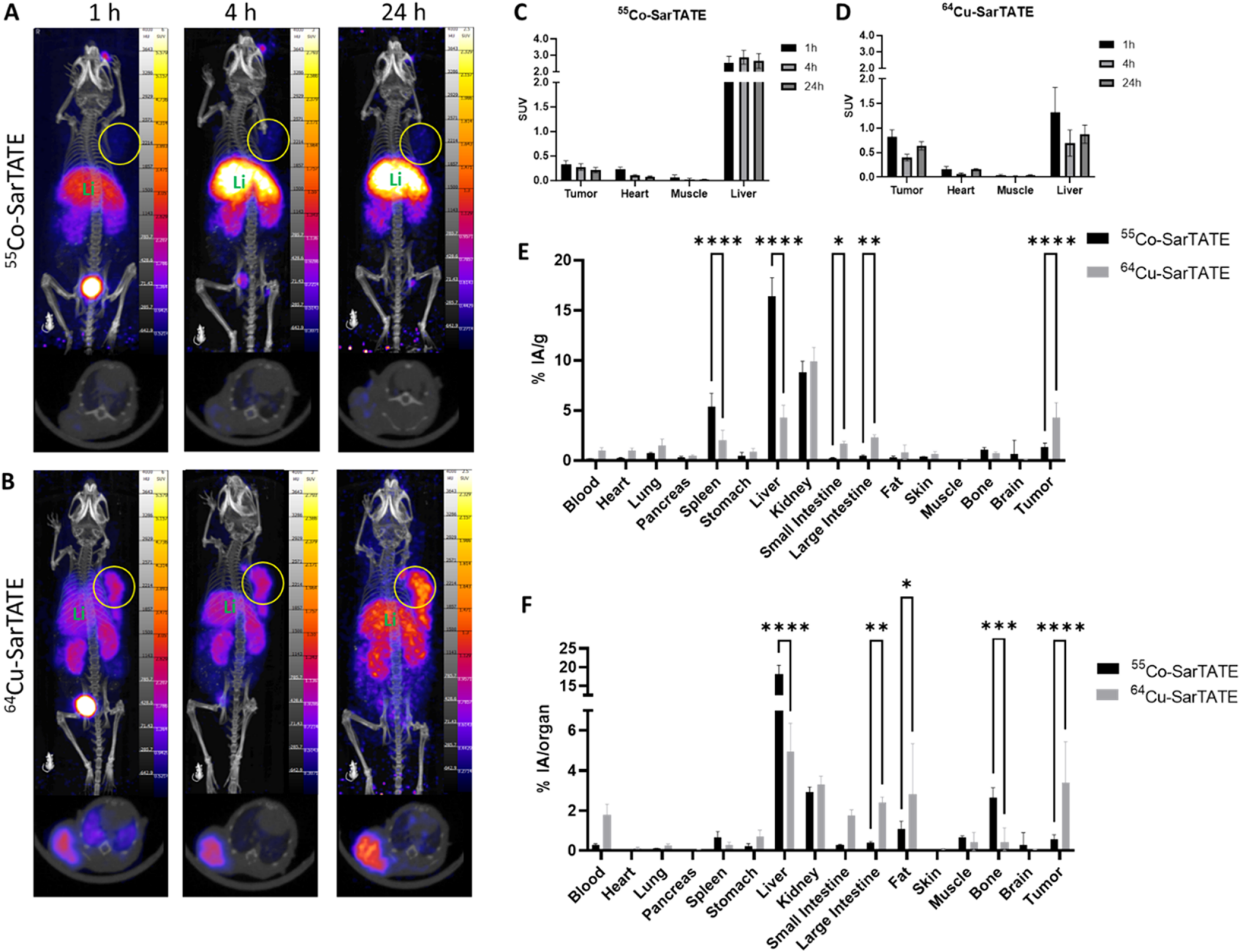
PET/CT imaging and biodistribution of [^55^Co]­[Co^III^(SarTATE)] and [^64^Cu]­[Cu^II^(SarTATE)] in AR42J tumor bearing mice. Mice injected with [^55^Co]­[Co^III^(SarTATE)] (A) or [^64^Cu]­[Cu^II^(SarTATE)] (B) were imaged up to 24 h post injection. Tumors are cicled in yellow and livers are annotated with “Li” in green. Images were processed to obtain SUV values (C, D). Biodistribution was evaluated for mice after the 24 h imaging time point and are expressed as % IA/g (E) and % IA/Organ (F).

In an effort to understand the difference in the distribution of [^55^Co]­[Co^III^(SarTATE)] and [^64^Cu]­[Cu^II^(SarTATE)] we investigated the distribution of [^55^Co]­[Co^III^(MeCOSar)] and [^64^Cu]­[Cu^II^(MeCOSar)] which both lack the targeting octreotate peptide. PET/CT images taken at 24 h post injection show the majority of the injected [^55^Co]­[Co^III^(MeCOSar)] was renally excreted and there is negligible apparent radioactivity in the liver. Additionally, compared to the distribution of [^55^Co]­[Co^II^Cl_2_] it appears that free cobalt is retained in many tissues including the blood, heart, liver, and kidneys (Figure 9A). Quantitatively, [^64^Cu]­[Cu^II^(MeCOSar)] and [^55^Co]­[Co^III^(MeCOSar)] behaved very similarly showing rapid clearance from all tissues (Figure 9B). Liver uptake of [^55^Co]­[Co^III^(SarTATE)] (16.40 ± 1.83%IA/g) was much higher than [^55^Co]­[Co^III^(MeCOSar)] (0.97 ± 0.23%IA/g) and [^55^Co]­[Co^II^Cl_2_] (4.53 ± 1.23%IA/g).

**Figure 9:**
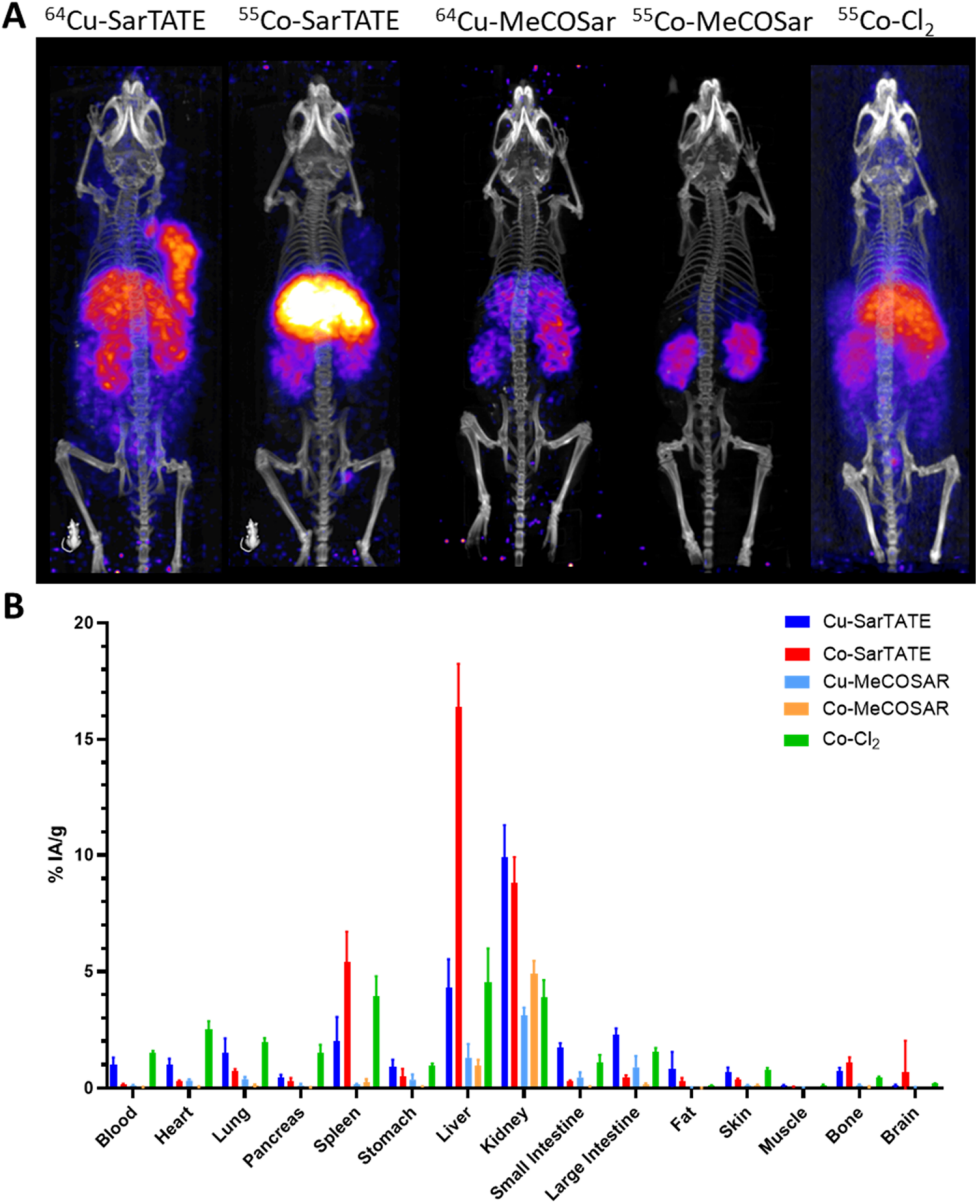
PET/CT images comparing 24 h distribution of SarTATE and MeCOSar, labeled with copper-64 and cobalt-55 with free [^55^Co]­CoCl_2_ (A). Biodistribution at 24 h post injection (B).

## Discussion

Characterization of [Co^III^(MeCOSar)]^3+^ by X-ray crystallography, NMR spectroscopy and electronic spectroscopy confirms the cobalt­(III) ion is a distorted octahedral environment as is characteristic of other cobalt­(III) complexes of substituted sarcophagines.^19^ Investigation of the electrochemical properties of the complex [Co^III^(MeCOSar)]^3+^ by cyclic voltammetry show that the complex undergoes a one electron quasi-reversible redox process consistent with reduction to a cobalt­(II) complex at *E*_1/2_ = −0.428 V vs SHE and this is likely to be outside biologically accessible redox potentials. It is also possible to chemically reduce the orange cobalt­(III) to a green cobalt­(II) complex using zinc as a reductant. This electrochemical behavior is similar to other cobalt­(III) sarcophagine complexes and suggests that complexation of MeCOSar and SarTATE with [^55^Co]­Co^II^ would initially result in the formation of the respective cobalt­(II) complexes which would be oxidized to their respective cobalt­(III) complexes by ambient concentrations of oxygen.^19^ The synthesis of [^55^Co]­[Co^III^(MeCOSar)]^3+^ and [^55^Co]­[Co^III^(SarTATE)] in radiochemical yields of >95% required a reaction mixture of pH ∼ 8 and heating to 80 °C whereas labeling both ligands with [^64^Cu]­Cu^II^ is possible at room temperature and pH ∼ 5. The fact that the rate of complexation of cobalt­(II) by sarcophagine ligands is slower than complexation of copper­(II) is consistent with an earlier report and the difference in ligand exchange kinetics between cobalt­(II) and copper­(II).^17^ In this work, [^55^Co]­[Co^III^(SarTATE)] was prepared at pH 8 and 80 °C in phosphate buffered saline to give [^55^Co]­[Co^III^(SarTATE)] with an apparent specific activity of 0.37 MBq/μg and an apparent molar activity of 0.54 MBq/nmol. It is possible that the presence of phosphate affects the complexation kinetics. A separate investigation, where a derivative of (NH_2_)_2_sar functionalized with a molecule designed to bind to the neurotensin receptor was radiolabeled with [^55^Co]­Co^III^ at pH 8 at 80 °C using HEPES buffer, achieved apparent molar activities of 9 MBq/nmol.^36^ It is possible that [^55^Co]­[Co^III^(SarTATE)] could be prepared in higher apparent molar activities if different buffers were used in place of phosphate buffered saline.

Previous studies demonstrated that [^55^Co]­Co^II^ and [^64^Cu]­Cu^II^ complexes of the DOTA derivative, DOTATATE^29^ have similar tumor uptake and biodistribution in somatostatin positive tumor models so the dramatically different tumor uptake and biodistribution between [^64^Cu]­[Cu^II^(SarTATE)] and [^55^Co]­[Co^III^(SarTATE)] (Figure 9) observed in this study was not expected. One notable difference between [^64^Cu]­[Cu^II^(SarTATE)] and [^55^Co]­[Co^III^(SarTATE)] is the different ionic charge between the metal ions (Cu^II^ and Co^III^). The ionic charge of the metal complex can have a dramatic effect on binding affinity, biodistribution and clearance.^29,33,34^ Furthermore, copper-64 and cobalt-55 complexes of NOTA or NODAGA chelators conjugated to the gastrin releasing peptide receptor targeting peptide RM26 showed similar tumor uptake between all tracers, but the Co-NOTA and Co-NODAGA compounds had significantly higher tumor to blood, tumor to liver, and tumor to lung ratios than their copper-64 labeled counterparts.^29^ Similarly, neurotensin targeting peptide NTS-20.3 linked to NOTA and radiolabeled with either cobalt-55 or copper-64 was compared in a colorectal cancer model demonstrated the highest tumor to heart ratio for [^55^Co]­Co-NOTA-NTS-20.3. In mice injected with [^64^Cu]­Cu-NOTA-NTS-20.3 a significant amount of copper-64 was observed in the liver by the 24 h imaging time point.^34^

One possible explanation of the high degree of radioactivity observed in the liver in the PET images following administration of [^55^Co]­[Co^III^(SarTATE)] could be release of [^55^Co]­Co^III/II^ from the ligand and subsequent accumulation of the ‘free’ [^55^Co]­Co^II^ in the liver. To test this possibility, we compared the biodistribution of [^55^Co]­[Co^III^(MeCOSar)]^3+^ and ‘free’ [^55^Co]­[Co^II^Cl_2_] in the same mouse model. The PET images 24 h post injection of [^55^Co]­[Co^III^(MeCOSar)]^3+^ show high uptake in the kidney and a distinctly different biodistribution to the images following injection [^55^Co]­[Co^II^Cl_2_], which results in significant activity in both the liver and kidneys. It is likely that the significant degree of uptake of activity in the liver following administration of [^55^Co]­[Co^III^(SarTATE)] reduces the amount of circulating tracer that is available to target the tumor. Furthermore, in mice that were injected with [^55^Co]­[Co^II^Cl_2_], liver uptake was much lower than what was observed following administration of [^55^Co]­[Co^III^(SarTATE)]. The high degree of kidney uptake and lack of background activity suggest that the [^55^Co]­[Co^III^(MeCOSar)]^3+^ is stable with respect to dissociation of the metal ion *in vivo*. At this stage, we are unable to explain the relatively high background signal following administration of [^55^Co]­[Co^III^(SarTATE)].

Additionally, we observed lower tumor uptake of [^64^Cu]­[Cu^II^(SarTATE)] than what was previously published.^25^ In the same AR42J xenograft model the tumor uptake 4 h after injection was 61.8 ± 2.4%IA/g, the kidney uptake was 16.0 ± 0.7%IA/g and lung uptake was 12.2 ± 0.8%IA/g.^25^ This is likely because the apparent molar activity of [^64^Cu]­[Cu^II^(SarTATE)] used in this work was lower than what was used in the previous study. This difference in apparent molar activity meant a higher mass dose of SarTATE was injected into each animal (1 μg compared to 0.35 μg) and this would likely lead to the partial blocking of some receptors. The elevated mass dose used in this study was necessary to better match the injected mass required to label SarTATE with cobalt-55.

## Conclusions

It is possible to prepare [^55^Co]­[Co^III^(MeCOsar)]^3+^ and [^55^Co]­[Co^III^(SarTATE)] in acceptable radiochemical yields but efficient radiolabeling requires heating to ∼80 °C. Evaluation of the biodistribution of [^55^Co]­[Co^III^(MeCOsar)]^3+^ revealed a high degree of activity in the kidneys with little to no activity in other organs and this is consistent with the hydrophilic complex retaining the metal ion in vivo. Surprisingly, evaluation of the tumor uptake of [^55^Co]­[Co^III^(SarTATE)] by PET imaging in a somatostatin positive tumor model, revealed relatively low tumor uptake and high background signal especially when compared to PET images acquired following administration of the copper­(II) complex, [^64^Cu]­[Cu^II^(SarTATE)]. Sarcophagine chelators favor the formation of kinetically inert d^6^ cobalt­(III) complexes, as opposed to relatively kinetically labile d^7^ cobalt­(II) complexes favored by DOTA and NOTA ligands, but the images acquired in somatostatin positive tumors in this study using [^55^Co]­[Co^III^(SarTATE)] have significantly lower tumor uptake than [^55^Co]­[Co­(DOTATATE)].^29^

## Supplementary Material



## Notes

Additional experimental details for the production of [^55^Co]­[CoCl_2_] and details for measuring the binding of [^55^Co]­[Co^III^(SarTATE)] and [^64^Cu]­[Cu^II^(SarTATE)]­binding AR42J cells (PDF)
