# Supplementary material for: An Evaluation of Cage Amine Sarcophagine Ligands for the Synthesis of Cobalt Radiopharmaceuticals
Source: Inorg Chem. 2026 May 27;65(22):12033–43. doi: 10.1021/acs.inorgchem.6c01482 (PMC13250976; doi:10.1021/acs.inorgchem.6c01482)
Supplement: Supplementary file 1 [file ic6c01482_si_001.pdf]

Supporting Information

An Evaluation of Cage Amine Sarcophagine Ligands for the Synthesis of Cobalt Radiopharmaceuticals

Hailey A. Houson[1], Stacey E. Rudd[2], Solana R. Fernandez[1], Suzanne E. Lapi[1*] and Paul S. Donnelly[2*]

[1]Department of Radiology, University of Alabama at Birmingham, Birmingham, AL 35294, USA.

[2]School of Chemistry and Bio21 Molecular Science and Biotechnology Institute, University of Melbourne, Parkville, Melbourne 3010, Australia. E-mail: pauld@unimelb.edu.au

E-mail: lapi@uab.edu; pauld@unimelb.edu.au

Production of [^{55}Co][$CoCl_2$]

Isotopically enriched nickel-58 (99.80%) was purchased from Isoflex (CA, USA) and was electroplated on a gold backing according to a previously published method [1]. To produce cobalt-55, the target was bombarded on an ACSI TR24 cyclotron at the UAB Cyclotron Facility for 3 hours at 18 MeV and 40 µA through a 0.75 mm aluminum degrader. After bombardment, the target was dissolved by heating at 80°C in 9 M HCl. The dissolved target was loaded onto a column with 2.5 g AG1x8 resin (Bio-Rad, CA, USA) and washed with 50 mL 9M HCl to remove nickel which was collected for recycling. Cobalt was eluted in 4 mL of 0.5 M HCl, redissolved in 9M HCL, and loaded onto a second 2.5 g AG1x8 resin column. The resin was washed again with 50 mL 9M HCl and cobalt was eluted in 2-2mL fractions of 0.5 M HCl. The second 2 mL fraction which contained the majority of the ^{55}Co was dried. Activity was reconstituted in 0.1 M HCl.

Effective molar activity (EMA) of the resulting cobalt-55 was assessed using 2,2′,2”,2”’-(1,4,7,10-tetraazacyclododecane-1,4,7,10-tetrayl)tetraacetic acid (DOTA) (Macrocyclics, Plano TX) by modification of published procedures.[1] Briefly, cobalt-55 was reconstituted in 0.5 M ammonium acetate buffer pH 5.5, combined with a serial dilution of DOTA and incubated at 50°C for 30 minutes. Samples were assessed for percent radiolabeling by spotting aluminum backed Si-60 plates and developing the plates in 0.5 M HCl. Plates were read using an AR-2000 Imaging Scanner (Eckert and Ziegler, MA, USA).

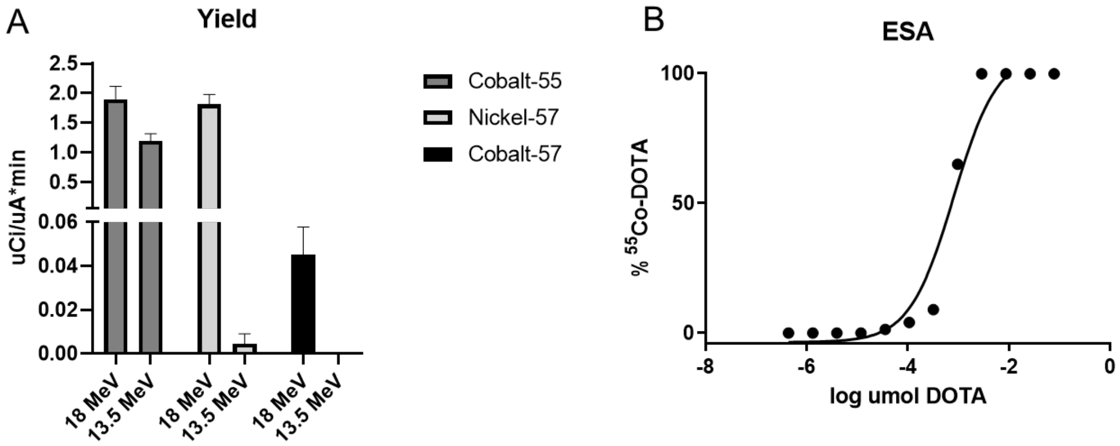

C

Metal concentration in ppb	Iron	Cobalt	Nickel	Copper
Cobalt-55	61000	64	574	16400
Copper-64	4800	19	13	1250

Figure S1: Production of ^{55}Co via the ^{58}Ni(p,α)^{55}Co production method. A) Yields of ^{57}Ni, ^{57}Co, and ^{55}Co are shown resulting from beam energy at 18 MeV and 13.5 MeV. B) An ESA curve from ^{55}Co produced at 13.5 MeV. C) Prominent metal contaminants in ^{55}Co-Cl$_2$ and ^{64}Cu-Cl$_2$ were determined using ICPMS.

Preparation of cobalt-55 *via* ^{58}Ni(p,α)^{55}Co at a proton energy of 18 MeV resulted in the production of nearly equivalent amounts of ^{57}Ni and ^{55}Co (70 and 66.9 kBq/μA*min respectively). In addition, cobalt-57 was also produced via the ^{58}Ni(p,2p)^{57}Co at a rate of 1.5 kBq/μA*min and is inseparable from cobalt-55 (Figure S1A). To reduce the formation of cobalt-57 the energy of the incident proton beam was reduced using a 0.75 mm aluminum degrader to 13.5 MeV. The lower energy reduced the yield of all three resulting isotopes to the extent that cobalt-57 became undetectable on the day of production (Figure S1A). The amount of nickel-57 produced also decreased to <0.4 kBq/uA*min allowing for more rapid recycling of the enriched target material. The average activity collected at the end of processing was 370 MBq. When estimated specific activity was assessed with DOTA, a typical result was 1.05 MBq/μmol (28.5 mCi/μmol) (Figure S1B). ICPMS was used to analyze the trace metal contaminants in cobalt-55 and copper-64 which determined that iron was the primary contaminant of cobalt (61000 ppb) and copper (4800 ppb) (Figure S1C).

Assessment of cell binding of metal complexes to AR42J cells

Gallium-68 was produced using a gallium generator (Eckert and Ziegler, Valencia, CA) by eluting the generator with 10 ml of 0.1 M HCl, trapping the [^{68}Ga][Ga^{III}] on a Phenomenex Strata-X-C 30 mg cartridge (Torrance, CA), and releasing the [^{68}Ga][Ga^{III}] in 400 μl of acetone. ([^{68}Ga][Ga^{III}(DOTATATE)] was produced by combining 32 μg of DOTATATE with the entire acetone eluate and 300 μl of 0.5 M sodium acetate pH 3.5 and heating at 98°C for 10 minutes. QC was performed using a one-to-one mixture of methanol and 10% W/V ammonium acetate in water using iTLC plates. Under these conditions free gallium remains at the baseline and [^{68}Ga][Ga^{III}(DOTATATE)] moves to the solvent front.

AR42J cells were obtained from ATCC (Manassas, VA, USA) and cultured in RPMI 1640 media containing 10% FBS and gentamycin (1 μg/mL) in a 5% CO_2 atmosphere. For binding studies, 250,000 cells were plated in 24 well plates and allowed to attach overnight. On the day of the assay, radiolabeled SarTATE and DOTATATE were incubated with the cells at indicated concentrations for 1 h at 37°C. At 1 hour, media was aspirated and cells were washed twice with 2 mL of cold PBS. Cells were lysed with 100 μL of 0.2 M NaOH and transferred to a tube for gamma counting. Samples and standards were counted on a gamma counter. Cell associated and standard counts were decay corrected and normalized to protein assayed by bicinchoninic acid assay (Pierce. ThermoFisher, Waltham, MA).

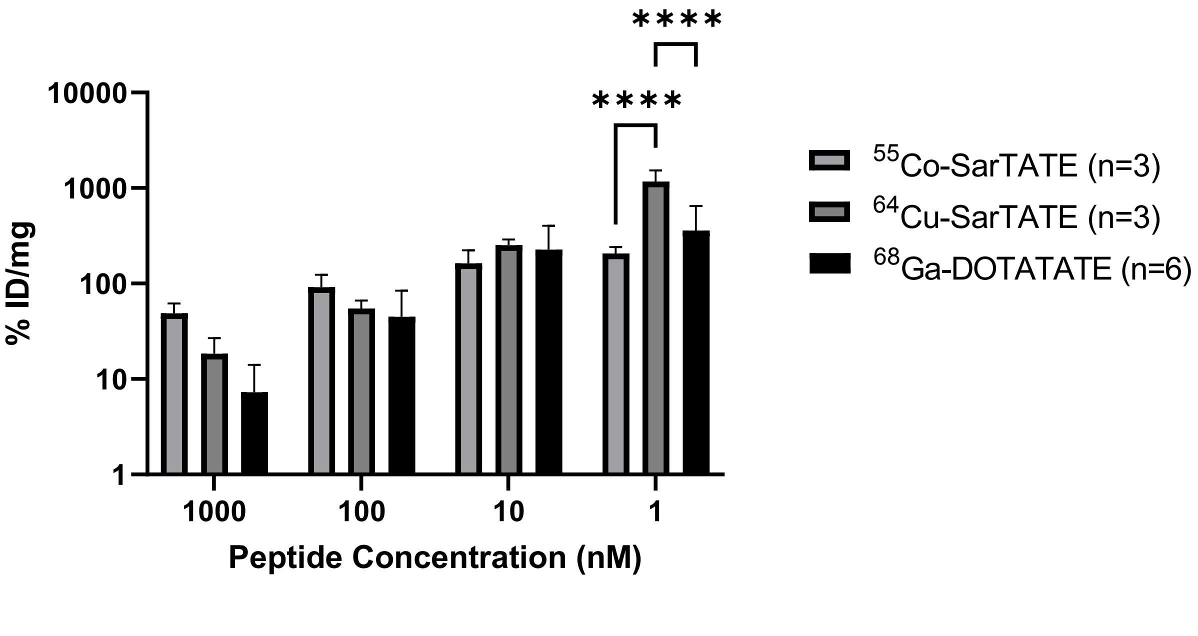

Figure S2: Cell uptake of SSTR2 binding peptides in AR42J cells.

Uptake of each tracer in was investigated in SSTR2 overexpressing AR42J cells. The uptake of [^{55}Co][Co^{III}(SarTATE)] (1 nM, 207 ± 34 % IA/mg) is similar to the uptake of [^{68}Ga][Ga^{III}(DOTATATE)] (1 nm, 360 ± 290 % IA/mg) and both were lower than uptake of [^{64}Cu][Cu^{II}(SarTATE)] (1 nm, 1200 ± 360 % IA/mg) (Figure S2).

References

1. Mastren, T.; Marquez, B. V.; Sultan, D. E.; Bollinger, E.; Eisenbeis, P.; Voller, T.; Lapi, S. E., Cyclotron Production of High-Specific Activity ^{55}Co and In Vivo Evaluation of the Stability of ^{55}Co Metal-Chelate-Peptide Complexes. *Mol Imaging* **2015,** *14* (10), 526-33.
